# The Effects of Cognitive-Affective Switching With Unpredictable Cues in Adults and Adolescents and Their Relation to “Cool” Executive Functioning and Emotion Regulation

**DOI:** 10.3389/fpsyg.2022.757213

**Published:** 2022-02-17

**Authors:** Jessica L. Samson, Lucien Rochat, Julien Chanal, Deborah Badoud, Nader Perroud, Martin Debbané

**Affiliations:** ^1^Developmental Clinical Psychology Research Unit, Faculty of Psychology and Educational Sciences, University of Geneva, Geneva, Switzerland; ^2^Service of Psychiatric Specialties, Department of Mental Health and Psychiatry, University Hospitals of Geneva, Geneva, Switzerland; ^3^Department of Psychiatry, University of Geneva, Geneva, Switzerland; ^4^Developmental Neuroimaging and Psychopathology Laboratory, Department of Psychiatry, University of Geneva School of Medicine, Geneva, Switzerland; ^5^Research Department of Clinical, Educational and Health Psychology, University College London, London, United Kingdom

**Keywords:** affective flexibility, cognitive-affective switching, hot and cool executive function, cognitive-emotion regulation, inhibition, inattention, adolescent/young adult literature

## Abstract

The impact of emotion on executive functioning is gaining interest. It has led to the differentiation of “cool” Executive Functioning (EF) processes, such as cognitive flexibility, and “hot” EF processes, such as affective flexibility. But how does affective flexibility, the ability to switch between cognitive and affective information, vary as a function of age and sex? How does this construct relate to “cool” executive functioning and cognitive-emotion regulation processes? In this study, 266 participants, including 91 adolescents (*M* = 16.08, SD = 1.42 years old) and 175 adults (*M* = 25.69, SD = 2.17 years old), completed a cognitive–affective switching task with specific (as opposed to general) unpredictable switches, as well as measures of inhibition, attention, and cognitive-emotion coping strategies. We expected cognitive to affective switching to be more costly than affective to cognitive switching in females versus males, as well as higher switch costs in adolescents. Using linear mixed modelling, we analysed the effect of age, sex, and types of switching on reaction time. Results show that adolescents are slower switchers than adults, and demonstrate that females, although faster switchers than males, are slower when switching from cognitive to affective content than when they are switching from affective to cognitive content. Multiple regression analyses revealed age-specific associations between cognitive-affective switching and inhibition. These results converge with reported developmental and gender specificities in EF and emotion processing, respectively. Additionally, affective flexibility could relate to differences in vigilance and inhibition.

## Introduction

Our daily occupations are rife with activities that entail switching between different tasks. Switching flexibly between different stimuli or tasks can be attributed to our ability for cognitive flexibility, a process involved in Executive Functioning (EF; Miyake et al., 2000; Ionescu, 2012; Diamond, 2013). However, EF is often at the mercy of emotion, which, by its very nature, tends to increase arousal and influence self-regulation of behaviour. As such, researchers now distinguish cognitive flexibility from *affective* flexibility (e.g., Kraft et al., 2020). Affective flexibility is that which allows us to switch to and from cognitive and affective information. This is understood under the conceptualisation that there are two categories of EF: “cool” EF, which refers to the traditional definition of EF, that allows for self-regulation of behaviour through the interaction between cognitive flexibility, inhibition and working memory, and “hot” EF, which refers to the goal-directed, future-oriented EF processes occurring in emotional and motivational contexts (Poon, 2018). The first usually involves logical thinking and controlled cognitive thoughts and actions, whereas the second involves affective processes, such as delaying instant gratification or affective decision-making (Zimmerman et al., 2016). Distinguishing both types of EF seems appropriate, given what is documented in the literature. For example, some studies suggest that “hot” and “cool” EF are independently affected in different clinical conditions, such as Attention Deficit Hyperactivity Disorder (ADHD) and Autism Spectrum Disorder (ASD; Antonini et al., 2015; Petrovic and Castellanos, 2016; Zimmerman et al., 2016). Moreover, even in non-clinical groups, affect states are known to disrupt “cool” cognitive processes and lead to incorrect or suboptimal choices in decision making, especially in adolescents (van Duijvenvoorde et al., 2010; Tsermentseli and Poland, 2016). In development, adolescence imposes rapid maturation both in the neurobiological and cognitive domains (Blakemore and Mills, 2014). Some processes, namely affective decision making, are even said to peak in mid-adolescence (14–16 years), thus increasing risk-taking behaviour (Casey et al., 2008; Casey, 2013; Poon, 2018). Furthermore, there is some evidence suggesting that, as opposed to “cool” EF, “hot” EF tends to develop in later adolescence, following the idea that EF processes become more distinct as age increases (Zelazo and Carlson, 2012). It is well established that the developmental events that occur in adolescence strike both the EF and emotion regulation domains (Zeman et al., 2006; Silvers et al., 2012; Blakemore and Mills, 2014). As such, in this study, we seek to examine age-related differences in affective flexibility and their relation to “cool” EF and emotion regulation in adolescents and young adults.

Flexibility is generally assessed using switching tasks, during which a participant must switch between two (or more) cognitive tasks (Allport et al., 1994; Rogers and Monsell, 1995). Switching results in a switch cost measured as an increase in reaction time (RT) or error rate. This process has been widely studied, demonstrating differences in switch costs across the lifespan (Cepeda et al., 2001) and in different clinical groups, such as ADHD (Cepeda et al., 2000; Kramer et al., 2001; McLean et al., 2004; White and Shah, 2006), Obsessive-Compulsive Disorder (Gu et al., 2008; Meiran et al., 2011), ASD (Stahl and Pry, 2002; Kleinhans et al., 2005; Schmitz et al., 2006; Shafritz et al., 2008; Poljac et al., 2010; Stoet and Lopez, 2011; de Vries and Geurts, 2012; Reed and McCarthy, 2012), and Schizophrenia (Posner et al., 1988; Nestor et al., 1992; Oie et al., 1998; Strauss et al., 2011).

On the other hand, affective flexibility has only been researched in a few studies using cognitive-affective switching tasks. The typical paradigm involves switching between identifying gender and emotion in faces of males and females depicting different emotions. In one study, participants switched between identifying colour, gender or emotion in happy or sad, male and female faces (Piguet et al., 2013). They found that identifying colour was less costly than identifying gender and emotion, but no difference was found between the two latter tasks. In a study conducted by Reeck and Egner (2015), they excluded the colour task condition, and found that switching from identifying gender to emotion was more costly than switching from emotion to gender. One reason for this effect may be explained by the sample used, which was only composed of women. However, the inconsistency in these studies’ results may also be explained by a difference in task configuration, whereby the task cues were predictable in the first study and unpredictable in the second. Considering that switching between affect and cognition is often of an unpredictable nature, we believe that a paradigm such as the one developed by Reeck and Egner is more ecologically valid and, thus, more suitable for the study of this construct.

In an earlier study, Reimers and Maylor (2005) examined affective flexibility across the lifespan, demonstrating that *general* (difference in performance between switching blocks and blocks with no switching at all), but not *specific* (difference in performance between switching and non-switching trials in a mixed switching block) switching costs decreased with age until 18 years and increased with age throughout adulthood. Once more, in their *specific* switching block, their task included a 1,000 ms Cue-Stimulus Interval (CSI), rendering the switches predictable, which may explain the lack of effect in the *specific* switching block. Furthermore, age differences using a non-affective switching paradigm with *specific* trials and unpredictable cues have been documented (Van Asselen and Ridderinkhof, 2000). However, the effects of an unpredictable cognitive-affective switching paradigm in adolescents and young adults have, to our knowledge, not been examined. Interestingly, sex differences were also found in the study conducted by Reimers and Maylor, revealing that in the *general* switching block when switching from a neutral to an affective task, switch costs were higher for females than males. This effect may support the reason why Reeck and Egner found a difference between cognitive to affective versus affective to cognitive switching costs in their study, as only women were tested. However, a confirmation of this effect in an unpredictable, *specific* (as opposed to *general*) switching paradigm should be confirmed when comparing both males and females.

The current study serves to improve our understanding of age and sex differences in affective flexibility and its relation to certain “cool” EF processes and cognitive-emotion regulation. As such, we developed a cognitive-affective switching task (CAST) with unpredictable cues (and thus, switches), similar to the one presented by Reeck and Egner (2015). Our objective is twofold. First, we wish to examine the age and sex differences in a *specific* and unpredictable cognitive-affective switching paradigm, using a sample of adolescents and young adults. Investigating affective flexibility in an unpredictable switching paradigm in adolescents would grant us an improved understanding of “hot” EF processes during this stage of life. Based on previous studies, we expect that cognitive to affective switching should be more costly than affective to cognitive switching (i.e., from affective to cognitive or vice versa), but only in women and that switch costs would differ as a function of age, whereby adolescents would be slower than adults. In addition, we test the impact of a double switch to gauge the residual impact of inhibition on RT. Finally, we investigate the link between the different types of switching costs (i.e., cognitive to affective or vice-versa) and inattention, inhibition and cognitive-emotion coping strategies. Concretely, we examine whether cognitive-affective switching costs would be related to higher scores of inattention, inhibition and “maladaptive” emotion regulation strategies. Investigating these leads would offer a first glimpse at how affective flexibility relates to “cool” EF and emotion regulation processes.

## Materials and Methods

### Participants

Typically developing (*N* = 266), French-speaking participants from the general population were recruited by undergraduate psychology students from the University of Geneva, Switzerland. This was done to complete an assignment for which the students received class credit. Non-native French speakers, psychology students, individuals that were below 12 or those over or equal to 31 years of age were excluded from this study. The students were trained beforehand to recognise this inclusion and exclusion criteria. Consent for adults was given by themselves and by parents or legal guardians for adolescents. Two groups were formed, distinguishing adults from adolescents: the first with 175 adults (94 female and 81 male) between 19.85 and 30.5 years old (*M* = 25.69, *SD* = 2.17), and the second with 91 adolescents (62 female and 29 male) between 13.02 and 18 years old (*M* = 16.08, *SD* = 1.42). These groups were formed on the basis of stark neurobiological and psychosocial differences that are present between these two age groups. Neurobiological arguments stress that, although developmental increases in “cool” executive control may be the result of a gradual maturation of prefrontal areas from childhood to early adulthood, there is a relative immaturity of prefrontal areas to the already mature affective-related limbic areas in adolescence specifically (Casey et al., 2008; Casey, 2013). Thus, the stage of maturation of affective/“hot” executive processes differentiate adolescents between 14 and 18 significantly from young adults. This is especially the case for affective decision making and motivation, which contribute in part to the increase in risk-taking behaviour at this age. Moreover, “hot” executive functioning, in particular affective flexibility, supports the ability to mentalise, which is itself developed mostly across adolescence and stabilises in early twenties (Blakemore and Mills, 2014). Therefore, it makes sense to differentiate adolescents from young adults in this manner, when investigating processes that support mentalisation, such as cognitive-affective switching (Bateman and Fonagy, 2012).

### Materials and Procedure

#### Cognitive-Affective Switching Task

This computerised task was designed to evaluate the ability to switch from processing affective content (emotion recognition task) to processing non-affective or cognitive content (gender recognition task). The stimuli employed were coloured pictures of male and female faces from the Karolinska Directed Emotional Faces (KDEF) database (Lundqvist et al., 1998). All faces modelled either happy or angry expressions and each face was presented within a green or blue frame which cued the task to be executed on every trial. Participants were told to respond as fast as possible without making mistakes, but disposed of an infinite amount of time to respond to each stimulus. There were six possible faces of each gender (male versus female) that could be presented. For each gender, three expressed anger and three expressed joy. The computer software suite E-Prime version 2.0 was used to program the task and for data collection (Psychology Software Tools, Inc, 2016, Pittsburgh, PA, United States).

Participants initiated the task with a training session of 16 trials. In the experimental session, there were 130 trials in total, 66 of which presented female faces, 33 expressing anger and 33 expressing joy, and 64 of which presented male faces, 32 expressing anger and 32 expressing joy. On half of the trials, the participants had to evaluate the emotion expressed on the face (affective task) and on the other half they had to evaluate the gender of the face (cognitive task). A blue framed stimulus cued the gender identification task, and a green framed stimulus cued the emotion identification task. Cue allocation was randomised and presented at the same time as the target rendering it unpredictable.

The participants were asked to select their answer as fast as possible using a standard computer QWERTZ keyboard. When evaluating gender, they tapped the letter “b” for man or “n” for woman, when evaluating emotion, they tapped the letter “x” for joy and “c” for anger. Participants were instructed to use their index and middle fingers on both hands so that all four keys were tapped with the same finger, and to avoid the switching of hands or fingers. The stimuli were presented following two fixed-order. A trial with a task (e.g., affective) could either be followed by a trial with the same task (i.e., affective, referring to a « control ») or by a trial with the alternative task (i.e., cognitive, referring to a « switch sequence »). In other words, in the switch sequence, the participants had to switch from an affective to a cognitive judgement (or the opposite). Whereas, in the control sequence they performed two affective (or two cognitive) judgements in a row. RT in milliseconds (ms) and correct responses were collected in each trial.

#### Visual Go-Nogo

This computerised task assesses automatic response inhibition and sustained attention (Robertson et al., 1997; Gay et al., 2008). The stimuli were 225 single digits (25 of each of the nine digits) presented visually one after the other during 258,000 ms (4.3 min). Each digit was presented for 250 ms followed by a 900 ms mask. Every digit onset was separated by a 1,150 ms interval. The participants were asked to press a key for every digit on the screen except when the digit 3 appeared. Mean reaction time (RT) in milliseconds, RT variability as measured by the standard deviation of RT divided by the mean RT (coefficient of variability; CV), percentage of commissions (an uninhibited key press when the number 3 appeared) and percentage of omissions (no key press for all other digits requiring one) were recorded. The computer software suite E-Prime 2.0 was also used to execute this task and collect data.

#### Questionnaire

A self-report questionnaire was used to assess emotion regulation processes, the French version of the Cognitive Emotion Regulation Questionnaire (CERQ; Jermann et al., 2006; d’Acremont and Van der Linden, 2007). The CERQ assesses “adaptive” and “maladaptive” cognitive-emotional regulation processes, namely acceptance, blaming others, self-blame, refocusing on planning, positive refocusing, dramatisation, positive reappraisal and rumination.

#### Procedure

Each participant was invited to the lab to perform all tests on the same day. They were first asked to provide consent. We began the session with the questionnaire and then participants performed the CAST, followed by the Go-Nogo. Both tasks were performed on the same computer.

### Statistical Analysis

Several analyses were performed using the open-source statistical programming software *R* (version: 1.2.5033; R Core Team, 2019).

#### Age and Sex Differences

Student *t*-tests were used to test for age and sex differences in the measure of the Go-Nogo and CERQ. These were corrected for multiple comparisons.

#### Cognitive-Affective Switching Effects and Group Differences in Switching

We constructed Linear Mixed Models (LMM) to compare the effects of the presence of a *n*-1 Switch (that is a switch occurring between the current trial *n* and the previous trial), the presence of a *n*-2 Switch (that is a switch occurring between the *n*-1 trial and the *n*-2 trial), the Task (emotion versus gender identification), Age (adult versus adolescent) and Sex (male or female) on RT at trial *n*, following a 2 × 2 × 2 × 2 × 2 factorial design. We included a subject random effect and an item random effect. The random effect structure had to be simplified in order to obtain convergence. Thus, we only included the random slope of the Switch *n*-1 variable. Lastly, our model controlled for the effects of trial number (Trial), the emotion depicted in the stimulus (EmoStim) and the sex of the person depicted in the stimulus (SexStim). To perform this analysis, we used the lmer function in the lme4 R package for LMM in combination with the afex package (for generating *p*-values which limit Type I errors; Bates et al., 2015; Singmann et al., 2017). This statistical method was used so as to include both the nested (multiple measurements within a single individual) and crossed (participants and stimuli) random structures of the data, providing accurate parameter estimates with acceptable type I error rates (Boisgontier and Cheval, 2016). As opposed to conventional analyses, such as the analysis of variance (ANOVA), these models also prevent the computation of RT means which retains the variability of responses in each condition and increases power (Judd et al., 2012).

#### Associations Between Switching Costs and Measures

Age- and gender-adjusted linear regressions were performed to test for associations between the different types of switch costs and the measures of the Go-Nogo and CERQ.

### Data Cleaning

Before constructing our LMM, corrected RTs of all variables were computed using RTs in correct trials only and trials not preceded by an error. This procedure was justified as error rates displayed a ceiling effect, with means between 0 and 10%. Furthermore, trials with RTs above or below 2.5 standard deviations (SD) of the participant’s mean were removed.

## Results

### Age and Sex Differences

The results of our *t*-tests (Table 1) revealed significant age differences for the coefficient of RT variability (CV), percentage of omissions and percentage of commissions in the Go-Nogo with higher values in the adolescent sample for all three variables. There were also significant differences for the Catastrophising, Blame-Other, Positive reappraisal and Refocus on planning dimensions of the CERQ, with higher values of the maladaptive processes in adolescents and higher values of the adaptive processes in adults.

**TABLE 1:** Descriptive statistics and corrected t-test results for age differences in measures of the Go-Nogo and CERQ.

		Adolescents		Adults				Adolescents + adults	
		Mean	SD	Mean	SD	*t*	*p*	Mean	SD
**Go-Nogo**	Mean RT	317.79	72.09	316.5	61.32	0.14512	0.88	316.94	65.07
	CV	0.34	0.18	0.27	0.12	3.1685	**0.001** **	0.3	0.15
	% omissions	3.87	5.99	1.79	3.16	3.0931	**0.02** *	2.5	4.44
	% commissions	51.86	22.02	42.94	22.65	3.1026	0.02*	45.99	22.79
**CERQ**	Self-blame	11.13	3.15	10.87	3.3	0.63752	0.53	10.96	3.25
	Rumination	13.18	3.45	12.61	3.9	1.2013	0.23	12.81	3.75
	Catastrophising	8.45	2.9	6.55	2.34	5.404	<0.0001***	7.2	2.7
	Blame other	8.43	2.65	7.3	2.25	3.4687	0.008**	7.69	2.45
	Acceptance	14.89	4.23	14.37	3.27	1.0169	0.31	14.55	3.63
	Positive refocusing	11.75	4.55	11.16	3.97	1.0388	0.30	11.36	4.18
	Positive reappraisal	13.54	3.12	15.45	3.36	−4.6046	0.0001***	14.79	3.4
	Refocus on planning	14.65	3.17	16.18	2.92	−3.8299	0.003**	15.65	3.09
	Putting into perspective	14.6	2.97	14.16	3.58	1.073	0.28	14.31	3.39

Bolded values are those that are statistically significant. The meaning of the asterisk signs are as follows: *p < 0.05, **p < 0.01, ***p < 0.001.

In terms of sex differences (Table 2), males and females differed significantly in the rumination dimension of the CERQ with higher values in females relative to males. All *p*-values were corrected for family-wise error rate using the Holm–Bonferroni sequential correction (Holm, 1979; Gaetano, 2018).

**TABLE 2:** Descriptive statistics and corrected t-test results for sex differences in measures of the Go-Nogo and CERQ.

		Female		Male				Female + male	
		Mean	SD	Mean	SD	*t*	*p*	Mean	SD
**Go-Nogo**	Mean RT	321.22	65.19	310.87	64.71	1.2804	0.20	316.94	65.07
	CV	0.3	0.15	0.29	0.14	0.2061	0.84	0.3	0.15
	% omissions	2.6	4.55	2.35	4.31	0.45176	0.65	2.5	4.44
	% commissions	43.61	21.62	49.37	24.05	−2.0039	0.48	45.99	22.79
**CERQ**	Self-blame	11.01	3.53	10.88	2.8	0.33892	0.73	10.96	3.25
	Rumination	13.56	3.63	11.73	3.68	3.9938	0.001**	12.81	3.75
	Catastrophising	7.32	2.86	7.03	2.45	0.8923	0.37	7.2	2.7
	Blame other	7.59	2.42	7.83	2.5	−0.76677	0.44	7.69	2.45
	Acceptance	14.31	3.9	14.89	3.18	−1.3196	0.19	14.55	3.63
	Positive refocusing	11.25	4.21	11.52	4.16	−0.52327	0.60	11.36	4.18
	Positive reappraisal	14.67	3.45	14.96	3.33	−0.688	0.49	14.79	3.4
	Refocus on planning	15.47	3.11	15.92	3.06	−1.1691	0.24	15.65	3.09
	Putting into perspective	14.33	3.44	14.29	3.32	0.079239	0.94	14.31	3.39

Bolded values are those that are statistically significant whether marginal or total. The meaning of the asterisk signs are as follows: *p < 0.05, **p < 0.01, ***p < 0.001.

### Cognitive-Affective Switching Effects and Group Differences in Switching

To investigate the contribution of each variable and their interaction, we compared the following models. We started with the simplest possible model (a model with only the controlled variables and random intercepts) and compared each model to ones of increasing complexity in a hierarchical fashion until the maximal model was reached. Model selection was based on the Akaike Information Criterion. All results are detailed in Tables 3–5.

**TABLE 3:** Linear Mixed Model results for models 0 to 6.

	Model 0	Model 1	Model 2	Model 3	Model 4	Model 5	Model 6	
**RT**								
**Fixed effects**				***b***				

Intercept	**1168.2466** ***	1042.9738***	998.2522***	961.5433***	1055.7274***	1078.7690***	1025.4510***	
Trial	−0.6452***	−0.7782***	−0.7721***	−0.8549***	−0.8545***	−0.8545***	−0.8833***	
SexStim	**−**18.1079	**−**18.1772	**−**18.0339	**−**19.3650	**−**19.3720	**−**19.3737	**−**19.1741	
EmoStim	**−**16.9978	**−**17.1153	**−**16.8884	**−**17.4588	**−**17.4658	**−**17.4645	**−**17.5340	
Switch *n*-1		261.9693***	261.7902***	262.8429***	262.8450***	262.8452***	262.4510***	
Emotion Task			89.8606***	89.8342***	89.8477***	89.8485***	90.1725***	
Switch *n*-2				79.7742***	79.7745***	79.7785***	78.7018***	
Age (adult)					−140.1818***	−144.0620***	−76.2956**	
Sex (female)						**−**35.1650	**−**19.4497	

**Random effects**				**σ**				***r***

**Subjects**								
Intercept	70757.0	70829.3	70737.3	70639.9	66304	66007	48602.2	
Switch *n*-1							18357.5	
Correlation (intercept, switch)								0.47
**Stimuli**								
Intercept	615.8	641.9	653.6	663.5	663	663	649.7	
Residual	203454.4	186168.7	184136.0	181503.1	181503	181503	176855.4	
Akaike Information Criterion	466641.1	463922.8	463588.2	461440.8	461426.4	461427.3	460908.9	

*p < 0.05, **p < 0.01, ***p < 0.001, ^[blank]^p > 0.1. Bolded values are those that are statistically significant.

**TABLE 4:** Linear Mixed Models results for models 7 to 14.

	Model 7	Model 8	Model 9	Model 10	Model 11	Model 12	Model 13	Model 14
**RT**								
**Fixed effects**				***b***				

Intercept	**1037.5947** ***	1041.0593***	1039.4363***	1060.5620***	1046.8531***	1036.5481***	1039.2948***	1044.2124***
Trial	−0.8843***	−0.8810***	−0.8812***	−0.8819***	−0.8840***	−0.8845***	**−**0.8841	−0.8839***
SexStim	**−**19.1363	**−**19.1363	**−**19.1521	**−**19.1368	**−**19.2406	**−**19.2555	**−**19.2628	**−**19.2607
EmoStim	**−**17.5828	**−**17.5532	**−**17.5430	**−**17.5334	**−**17.5611	**−**17.5794	−17.5807***	**−**17.5791
Switch *n*-1	237.6041***	230.5436***	230.6347***	278.1526***	278.8797***	278.7585***	278.7777***	289.6396***
Emotion Task	65.2567***	65.1906***	68.4422***	68.3930***	68.3806***	89.3695***	83.7849****	83.7639***
Switch *n*-2	78.6001***	71.4508***	74.6681***	74.7013***	102.5096***	102.7138***	102.6786***	102.6530***
Age (adult)	−76.0211**	−76.0562**	−76.0371**	−107.6144***	−87.1677**	−71.9118*	−72.4225*	−73.2470*
Sex (female)	**−**19.3660	**−**19.2976	**−**19.3018	**−**19.2475	**−**19.3565	**−**19.3916	**−**23.5512	**−31.0652**
Switch *n*-1 × Emotion Task	50.2355***	50.3130***	50.1444***	50.2164***	50.2149***	50.2222***	50.2183***	50.2213***
Switch *n*-1 × Switch *n*-2		14.4473	14.4587	14.4546	14.4660	14.4661	14.4499	14.4691
Switch *n*-2 × Emotion Task			**−**6.5141	**−**6.5344	**−**6.4045	**−**6.4235	**−**6.4654	**−**6.4584
Switch *n*-1 × Age				−70.5383***	−71.6198***	−71.4938***	−71.4933***	−73.3385***
Switch *n*-2 × Age					−40.9591***	−41.1917***	−41.1055***	−41.0922***
Emotion Task × Age						−30.8468**	−29.8726**	−29.8695**
Emotion Task × Sex							8.4729	8.4881
Switch *n*-1 × Sex								**−**16.5516
Switch *n*-1 × Emotion Task × Sex								

**Random effects**	**σ**							

**Subjects**								
Intercept	48669.0	48688.0	48692.7	48483.0	48446.1	48424.2	48429.1	48688.0
Switch *n*-1	18327.9	18328.9	18321.7	17235.6	17212.2	17222.2	17217.9	18328.9
**Stimuli**								
Intercept	648.2	648.9	649.2	649.7	654.2	654.4	655.6	648.9
Residual	176695.5	176681.3	176679.0	176677.8	176590.3	176538.2	176533.8	176681.3
Akaike Information Criterion	460883.5	460883.3	460884.8	460874.7	460860.9	460853.9	460855.2	460858.4

*p < 0.05, **p < 0.01, ***p < 0.001, ^[blank]^p > 0.1. Bolded values are those that are statistically significant.

**TABLE 5:** Linear Mixed Model results for final model 15.

	Model 15				

**RT**				**Confidence intervals**	
**Fixed effects**	***b***	**SE**	***p***	**Low**	**Up**
Intercept	1036.4646	34.6466	<0.0001***	968.56	1104.4
Trial	**−**0.8841	0.1295	<0.0001***	**−**1.14	**−**0.63
SexStim	**−**19.2413	15.5533	0.24	**−**49.723	11.24
EmoStim	**−**17.5947	15.5534	0.28	**−**48.08	12.89
Switch *n*-1	305.4129	22.4174	<0.0001***	261.48	349.35
Emotion Task	99.5864	13.8068	<0.0001***	72.53	126.65
Switch *n*-2	102.6012	10.8428	<0.0001***	81.35	123.85
Age (adult)	**−**73.2767	30.7120	0.02*	**−**133.47	**−**13.08
Sex (female)	**−**17.6794	29.1878	0.55	**−**74.89	39.53
Switch *n*-1 × Emotion Task	18.2530	14.8535	0.22	**−**10.86	47.37
Switch *n*-1 × Switch *n*-2	14.4426	9.6372	0.13	**−**4.45	33.33
Switch *n*-2 × Emotion Task	**−**6.3957	9.6319	0.51	**−**25.27	12.48
Switch *n*-1 × Age	**−**73.2799	20.1083	0.0003**	**−**112.69	**−**33.87
Switch *n*-2 × Age	**−**41.0851	10.3148	<0.0001***	**−**61.30	**−**20.87
Emotion Task × Age	**−**29.8738	10.3356	0.004*	**−**50.13	**−**9.62
Emotion Task × Sex	**−**18.6768	13.7319	0.17	**−**45.59	8.24
Switch *n*-1 × Sex	**−**43.6347	21.3855	0.04*	**−**85.55	**−**1.72
Switch *n*-1 × Emotion Task × Sex	54.7844	19.4407	0.005**	16.68	92.89
**Random effects**					
**Subjects**					
Intercept	48400.1				
Switch	17170.8				
**Stimuli**					
Intercept	656.8				
Residual	176486.6				
Akaike Information Criterion	460850.5				

Bolded values are those that are statistically significant. *p < 0.05, **p < 0.01, ***p < 0.001.

#### Model 0

Model 0 includes the random intercepts and our controlled variables. The effect of Trial was significant [β = −0.6452, SE = 0.1382, *t*(30642.6223) = −4.668, *p* < 0.000, *R*^2^ marginal = 0.1% *R*^2^ conditional = 26%] showing that RT tends to decrease with increasing trials and that the emotion (EmoStim) and the sex (SexStim) depicted in the stimuli do not affect RT.

#### Model 1

We added the effect of a *n*-1 Switch on RT at trial *n*. The effect was significant [β = 261.9693, SE = 4.9117, *t*(30641.4833) = 53.336, *p* < 0.000, *R*^2^ marginal = 6.3% *R*^2^ conditional = 32%] suggesting that RT is increased by 261 ms in trial *n* (i.e., the current trial) when there is presence of a switch versus the absence of a switch between trials *n* and *n*-1 (i.e., between the current and previous trial). All other effects remained unchanged. The Akaike Information Criterion (AIC) indicates that model 1 (AIC = 463922.8) estimates the data more accurately than model 0 (AIC = 466641.1).

#### Model 2

We added the effect of the emotion identification task (Emotion Task) on RT at trial *n*. The effect was significant (β = 89.8606, SE = 4.8845, *t*(30641.2139) = 18.397, *p* < 0.000, *R*^2^ marginal = 7% *R*^2^ conditional = 33%) suggesting that RT is increased by 89 ms at trial *n* when the task to be performed is the emotion identification task versus the gender identification task. All other effects remained unchanged. The Akaike Information Criterion indicates that model 2 (AIC = 463588.2) estimates the data more accurately than model 1 (AIC = 463922.8).

#### Model 3

We added the effect of a *n*-2 Switch on RT at trial *n*. The effect was significant [β = 79.7742, SE = 4.8657, *t*(30527.1621) = 16.395, *p* < 0.000, *R*^2^ marginal = 7.6% *R*^2^ conditional = 33.7%], suggesting that RT in trial *n* (i.e., the current trial) is increased by 79 ms when there is presence of a switch versus the absence of a switch between trials *n*-1 and *n*-2 (i.e., the effect of a switch between the previous trial and the one before that on RT in the current trial). All other effects remained unchanged. The Akaike Information Criterion indicates that model 3 (AIC = 461440.8) estimates the data more accurately than model 2 (AIC = 463588.2).

#### Model 4

We added the effect of Age (as a group) on RT. The effect was significant [β = −140.1818, SE = 34.0863, *t*(265.0943) = −4.113, *p* < 0.000, *R*^2^ marginal = 9.2% *R*^2^ conditional = 33.7%], suggesting that RT is 140 ms shorter in adults versus adolescents. All other effects remained unchanged. The Akaike Information Criterion indicates that model 4 (AIC = 461426.4) estimates the data more accurately than model 3 (AIC = 461440.8).

#### Model 5

We added the effect of Sex on RT. The effect was non-significant [β = −35.1650, SE = 32.5474, *t*(264.9483) = −1.080, *p* = 0.28, *R*^2^ marginal = 9.3% *R*^2^ conditional = 33.7%], suggesting that RT does not vary significantly between males and females. All other effects remained unchanged. The Akaike Information Criterion indicates that model 5 (AIC = 461427.3) does not estimate the data more accurately than model 4 (AIC = 461426.4). However, we kept the sex variable in the model in order to test for interactions in later models.

#### Model 6

Here, we allowed the effect of *n*-1 Switch to vary randomly among participants. This not only significantly improved the model fit, as can be seen by the improved Akaike Information Criterion (AIC = 460908.9; *R*^2^ marginal = 8.2% *R*^2^ conditional = 34.8%) relative to model 5 (AIC = 461427.3). but it also decreased the subject explained variance from 66007 to 48602.2, with a variance explained of 18357.5 by the *n*-1 Switch random effect. Moreover, the speed of an individual is positively correlated (*r* = 0.47) with the variability of the *n*-1 Switch effect, suggesting that slower individuals are also more variable in the presence of a switch. All other effects remain significant. The magnitude of the Age effect is slightly lowered suggesting that the random effect of the *n*-1 Switch helps explain some of the variability due to age differences.

#### Model 7

We added the Emotion Task × *n*-1 Switch interaction. The effect was significant [β = −50.2355, SE = 9.5908, *t*(30289.7909) = 5.238, *p* < 0.000, *R*^2^ marginal = 8.2% *R*^2^ conditional = 34.9%], suggesting that the effect of *n*-1 Switch is increased by 50 ms during the emotion task versus the gender task. Therefore, switching from gender to emotion is more costly than switching from emotion to gender. All other effects remained unchanged. The Akaike Information Criterion indicates that model 7 (AIC = 460883.5) estimates the data more accurately than model 6 (AIC = 460908.9).

#### Model 8

We added the *n*-1 Switch × *n*-2 Switch interaction. The effect was non-significant [β = −14.4473, SE = 9.6425, *t*(30300.8763) = 1.498, *p* = 0.13, *R*^2^ marginal = 8.2% *R*^2^ conditional = 34.9%], suggesting that the effect of a *n*-1 Switch on RT in trial *n* does not vary as a function of the presence of *n*-2 Switch. Therefore, switching twice in a row does not differ compared to switching only once. All other effects remained unchanged. The Akaike Information Criterion indicates that model 8 (AIC = 460883.3) estimates the data only slightly more accurately than model 7 (AIC = 460883.5), but the difference is non-significant.

#### Model 9

We added the *n*-2 Switch × Emotion Task interaction. The effect was non-significant [β = −6.5141, SE = 9.6370, *t*(30306.2129) = −0.676, *p* = 0.50, *R*^2^ marginal = 8.2% *R*^2^ conditional = 34.9%], suggesting that the effect of a *n*-2 Switch on RT in trial *n* does not vary as a function of the task being performed. All other effects remained unchanged. The Akaike Information Criterion indicates that model 9 (AIC = 460884.8) does not estimate the data more accurately than model 8 (AIC = 460883.3).

#### Model 10

**FIGURE 1:**
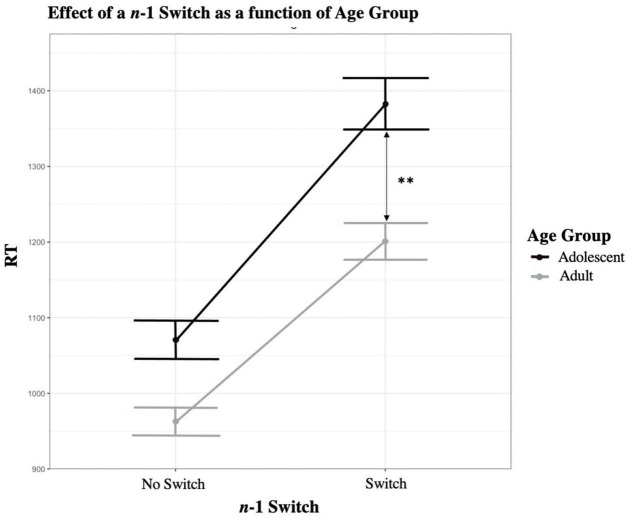
Effect of a *n*-1 Switch as a function of age group. Graphical representation of the *n*-1 Switch × Age interaction. The difference in RT when there is a *n*-1 Switch versus no switch is significantly greater in adolescents versus adults. *p < 0.05, **p < 0.01, ***p < 0.001.

We added the *n*-1 Switch × Age interaction (see Figure 1). The effect was significant [β = −70.5383, SE = 20.0240, *t*(268.0001) = −3.523, *p* < 0.001, *R*^2^ marginal = 9.4% *R*^2^ conditional = 35.4%], suggesting that the effect of *n*-1 Switch on RT in trial *n* is 71 ms lower in adults versus adolescents. All other effects remained significant. The age effect slightly improved in magnitude, suggesting that adding this interaction improves the variance explained by the effect of age. The Akaike Information Criterion indicates that model 10 (AIC = 460874.7) estimates the data more accurately than model 9 (AIC = 460884.8).

#### Model 11

We added the *n*-2 Switch × Age interaction. The effect was significant [β = −40.9591, SE = 10.3171, *t*(30463.9506) = −3.970, *p* < 0.000, *R*^2^ marginal = 9.4% *R*^2^ conditional = 35.4%], suggesting that the effect of *n*-2 Switch on RT in trial *n* is 40 ms lower in adults versus adolescents. All other effects remained significant. The age effect lessened slightly in significance, suggesting that this interaction helps explain some of the variability in the effect of age on RT. The Akaike Information Criterion indicates that model 11 (AIC = 460860.9) estimates the data more accurately than model 10 (AIC = 460874.7).

#### Models 12

We added the Emotion Task × Age interaction. The effect was significant [β = −30.8468, SE = 10.2757, *t*(30288.9377) = −3.002, *p* = 0.002, *R*^2^ marginal = 9.4% *R*^2^ conditional = 35.4%], suggesting that the effect of the emotion identification on RT in trial *n* is 30 ms lower in adults versus adolescents. The age effect is slightly lessened in significance once more, suggesting that this interaction helps explain some of the variability in the effect of age on RT. The Akaike Information Criterion indicates that model 12 (AIC = 460853.9) estimates the data more accurately than model 11 (AIC = 460860.9).

#### Model 13

We added the Emotion Task × Sex interaction. The effect was non-significant [β = 8.4729, SE = 9.7810, *t*(30288.8622) = 0.866, *p* = 0.39, *R*^2^ marginal = 9.4% *R*^2^ conditional = 35.4%], suggesting that the effect of the emotion task on RT in trial *n* does not vary as a function of age. All other effects remained unchanged. The Akaike Information Criterion indicates that model 13 (AIC = AIC = 460855.2) does not estimate the data more accurately than model 12 (AIC = 460853.9).

#### Model 14

We added the *n*-1 Switch × Sex interaction. The effect was non-significant [β = −16.5516, SE = 19.0984, *t*(266.4180) = −0.867, *p* = 0.39, *R*^2^ marginal = 9.5% *R*^2^ conditional = 35.4%], suggesting that the effect of the *n*-1 Switch on RT in trial *n* does not vary as a function of age. All other effects remained unchanged. The Akaike Information Criterion indicates that model 14 (AIC = 460858.4) does not estimate the data more accurately than model 13 (AIC = 460855.2).

#### Model 15

**FIGURE 2:**
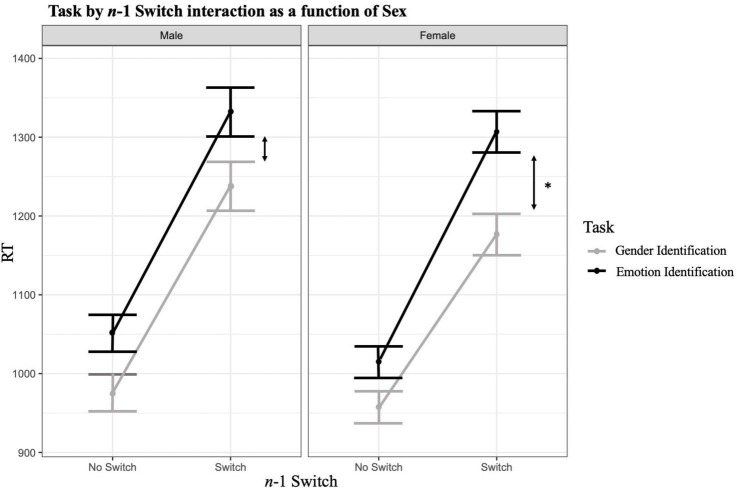
Task by *n*-1 Switch Interaction as a function of sex. Graphical representation of the *n*-1 Switch × Task Emotion × Sex interaction. The difference in RT when there is an *n*-1 Switch × Emotion Task interaction is more pronounced in females versus males. *p < 0.05, **p < 0.01, ***p < 0.001.

Finally, we added the *n*-1 Switch × Task Emotion × Sex interaction. This effect was significant [β = −40.9591, SE = 10.3171, *t*(30463.9506) = −3.970, *p* < 0.000, *R*^2^ marginal = 9.5% *R*^2^ conditional = 35.5%], suggesting that the increase in RT when there is presence of a switch and the emotion task is increased by 54 in females relative to males. By adding this effect, the *n*-1 Switch × Sex interaction becomes significant, suggesting the effect of a *n*-1 Switch on RT in trial *n* is decreased by 43 ms in females relative to males. On the other hand, the *n*-1 Switch × Task Emotion interaction becomes non-significant, suggesting that this effect was driven by one of the two sex groups and is only present in females. The combination of these effects implies that, although females are generally faster switchers than males when switching from gender to emotion, the cost difference in RT between switching from gender to emotion than from emotion to gender is greater in females than it is in males (see Figure 2). The Akaike Information Criterion indicates that model 15 (AIC = 460850.5) estimates the data more accurately than model 14 AIC = 460858.4) and all previous models. All other three-way or four-way interactions were tested as well, but none reached significance. One of these four-way interactions tested whether there was a difference in the *n*-1 Switch × Task Emotion × Sex interaction as a function of Age. This effect was non-significant indicating that the male and female difference found above did not differ between adolescents and adults. We conclude that model 15 is the best fitting model.

#### Regression Analysis

In the adolescent and adult combined group, multiple linear regressions adjusted for age and sex were computed to assess associations between all types of switch costs and all variables of the Go-Nogo and CERQ. Holm–Bonferroni sequential correction was applied to account for family-wise error rate. Results of the combined switch costs (emotion to gender and gender to emotion switches combined) showed a positive association with mean RT (β = 0.8296, *p* < 0.000), percentage of omissions (β = 13.6083, *p* = 0.01) and percentage of commissions (β = 1.9001, *p* = 0.006) in the Go-Nogo. Only the association with mean RT survived correction. An association with the putting into perspective dimension of the CERQ reached marginal significance (β = 5.6441, *p* = 0.09). Only the association with mean RT survived correction in this model and the model itself was significant [*R*^2^ = 0.17, adjusted *R*^2^ = 0.12, *F*(15,75) = 3.473, *p* < 0.000]. It was also found that emotion to gender switch costs were positively associated with mean RT (β = 0.54564, *p* = 0.02) and percentage of omissions (β = 16.60160, *p* < 0.000) in the Go-Nogo, as well as with the blame-other dimension of the CERQ (β = 10.44126, *p* = 0.04). However, only the relationship with the percentage of omissions survived correction. A relationship of marginal significance between emotion to gender switch costs and the RT coefficient of variability (CV; β = −260.46521, *p* < 0.000) was found as well, which did not survive correction, and the overall model was significant [*R*^2^ = 0.12, adjusted *R*^2^ = 0.07, *F*(15,75) = 2.318, *p* = 0.004]. On the other hand, gender to emotion switch costs were positively associated with mean RT (β = 1.11396, *p* < 0.000) and percentage of commissions (β = 2.71133, *p* = 0.001) in the Go-Nogo. Both these effects survived correction. An association with the putting into perspective dimension of the CERQ also reached marginal significance (β = 7.04986, *p* = 0.08), but did not survive correction. The overall model for gender to emotion switching in the combined sample was significant [*R*^2^ = 0.17, adjusted *R*^2^ = 0.12, *F*(15,75) = 3.465, *p* < 0.000].

In the adolescent sample alone, combined switch costs were significantly associated with percentage of omissions (β = 29.8943, *p* < 0.000) and CV (β = −748.9218, *p* = 0.01). A relationship of marginal significance was found with the blame-other dimension of the CERQ (β = 15.7896, *p* = 0.07). However, only the relationship with percentage of omissions survived correction, with the overall model being significant [*R*^2^ = 0.29, adjusted *R*^2^ = 0.15, *F*(15,75) = 2.032, *p* < 0.02]. Emotion to gender switch costs were positively associated with percentage of omissions (β = 26.33421, *p* = 0.004), and marginally with CV (β = −533.09301, *p* = 0.08) of the Go-Nogo and the blame-other (β = 18.41550, *p* = 0.05) dimension of the CERQ. Only the relationship with percentage of omissions survived correction. The overall model for emotion to gender switching was marginally significant [*R*^2^ = 0.24, adjusted *R*^2^ = 0.10, *F*(15,75) = 1.581, *p* = 0.09]. On the other hand, gender to emotion switch costs were positively associated with mean RT (β = 0.9973, *p* = 0.04), CV (β = −973.0920, *p* = 0.009), percentage of omissions (β = 33.7646, *p* = 0.002) and percentage of commission errors (β = 4.1206, *p* = 0.03) in the Go-Nogo. Once more, only the relationship percentage of omissions survived correction. The overall model was significant [*R*^2^ = 0.27, adjusted *R*^2^ = 0.13, *F*(15,75) = 1.877, *p* < 0.04].

In the adult sample alone, combined switch costs were positively associated with mean RT (β = 1.0451, *p* < 0.000) and percentage of commissions (β = 2.1686, *p* = 0.005) in the Go-Nogo, both of which survived correction. The overall model was significant [*R*^2^ = 0.18, adjusted *R*^2^ = 0.10, *F*(15,75) = 2.267, *p* < 0.001]. Emotion to gender switch costs were positively associated with mean RT (β = 0.7931, *p* = 0.02), percentage of commissions (β = 1.8916, *p* = 0.04) and marginally with percentage of omissions (β = 11.5486, *p* = 0.06) in the Go-Nogo. None of this model’s associations survived correction. The overall model for this type of switching was not significant [*R*^2^ = 0.09, adjusted *R*^2^ = 0.01, *F*(15,75) = 1.005, *p* = 0.45]. On the other hand, gender to emotion switch costs were positively associated with mean RT (β = 1.2780, *p* < 0.000), percentage of commissions (β = 2.3951, *p* = 0.005) in the Go-Nogo and the acceptance dimension (β = 9.9232, *p* = 0.02) of the CERQ. The latter did not survive correction. The overall model for gender to emotion switching in adults was significant [*R*^2^ = 0.18, adjusted *R*^2^ = 0.10, *F*(15,75) = 2.267, *p* < 0.001].

## Discussion

This study was conducted in an attempt to better our understanding of developmental and sex differences in affective flexibility, and to unravel the potential associations between affective flexibility, “cool” EF, and emotion regulation (ER). We were particularly interested in the differences in affective flexibility between adolescents and young adults, as well as how cognitive-affective switching costs in a paradigm with unpredictable cues could relate to inattention, inhibition and cognitive-emotion regulation processes. We present a first attempt at relating these constructs providing new findings on developmental specificities on associations between affective flexibility, “cool” EF and emotion regulation, as well as new information regarding sex differences in *specific* (as opposed to general) unpredictable cognitive-affective switching. We have structured our discussion to include the conclusions of our results, starting with our descriptive analysis, followed by our LMM analysis and ending with our regression analysis. We conclude with a section on limitations and future directions.

### Age and Sex Differences in the Go-Nogo and the Cognitive Emotion Regulation Questionnaire

Our first analyses revealed age differences in inhibition and emotion regulation. Specifically, adolescents tended to perform more commission and omission errors and tended to have more variable reaction times in the Go-Nogo. This is consistent with the literature indicating developmental differences in executive functioning (Blakemore and Mills, 2014). These differences go hand-in-hand with the development of emotion regulation processes, showing that emotion regulation evolves and improves during adolescence (Zeman et al., 2006; Silvers et al., 2012). Indeed, the adolescents in our sample seem to be more prone to catastrophise and blame others and less prone to refocus on planning and reappraise positively. The only significant result in sex comparisons was a difference in rumination scores, which is consistent with the literature suggesting higher rumination tendencies in females compared to males (Johnson and Whisman, 2013 for a meta-analysis).

### Cognitive-Affective Switching

Our mixed model analysis shows that our cognitive-affective switching task (CAST) does indeed measure switching costs when switching to and from cognitive and affective content. We expected that switching would be more costly for adolescents than for adults. Our results demonstrate that adolescents tend to be slower switchers than adults. In line with the results of Reeck and Egner’s study, the emotion task tends to have a general slowing effect on RT relative to the gender task. This is unsurprising seeing as an emotional task is associated with a dominant task-set and attentional capture is generally biassed towards affective stimuli (Monsell et al., 2000; Bar-Haim et al., 2007; Reeck and Egner, 2015; Pool et al., 2016). We also expected to find that cognitive to affective switching would be more costly than affective to cognitive switching. We found this result to be specific to the female group as expected. Previous studies reported sex differences in switching as well as a switch × task interaction, whereby cognitive to affective switching is more costly than affective to cognitive switching in females (Reimers and Maylor, 2005; Reeck and Egner, 2015). These results were not replicated in models 7 to 14. However, as soon as we added a switch × task × sex interaction, both effects reversed, showing a significant switch × sex interaction and a non-significant switch × task interaction. This suggests that females, although faster switchers than males when switching from affective to cognitive content, may be more impacted than males by the affective stimuli in the task, which slows their ability to switch from cognitive to affective relative to their ability to switch from affective to cognitive content. The idea that females may be more affected by emotional material could partly be explained by a higher degree of emotional reactivity or arousal towards emotional stimuli (Bradley et al., 2001; Koch et al., 2007; Rueckert et al., 2011; Bianchin and Angrilli, 2012). Indeed, several studies show increased emotional reactivity in females, especially towards negative stimuli. It has also been suggested that females are more susceptible to emotional contagion (Doherty et al., 1995; Wild et al., 2001). Some studies have even reported more pronounced attentional capture towards affective stimuli in females compared to males (Sass et al., 2010; Pfabigan et al., 2014). This opens questions on how affective flexibility and other “hot” EF constructs differ in clinical populations that are particularly characterised by emotional reactivity such as BPD and ADHD. Indeed, it has already been shown that using an emotional variant of the Go-Nogo in these clinical groups provides differences in inhibiting responses to affective stimuli (Köchel et al., 2014; Sinke et al., 2017). Researching these topics could provide insight on how affective switching is associated with symptoms of emotional dysregulation in clinical populations.

The age differences in this task do not reflect age-specific differences related to cognitive-affective switching *per se*, but may be more generally related to differences in “cool” EF and inhibition of affective stimuli in general. Indeed, our results show that the adolescent sample seems to be more slowed by the affective stimuli in this task than the adult sample, but that this is the case regardless of whether they are asked to switch or not. Adolescents show no significant difference compared to adults between when they switch from cognitive to affective information and affective to cognitive information. However, they are slower than adults when performing the task in general and they also switch more slowly than adults. Their reaction time in trial *n* is also more affected when they performed a switch between trial *n*-2 and *n*-1, when there is absence of a switch between trials *n*-1 and *n*. These results suggest that age related differences in affective flexibility reflect primarily differences in “cool” EF processes, namely cognitive flexibility and possibly the ability to inhibit affective stimuli in general. The latter conclusion is supported by one study that has shown effects in an emotional go-nogo task that are specific to adolescents relative to children and adults, whereby adolescents responded faster to emotional “go” trials than neutral “go” stimuli compared to children and adults, but male adolescents were penalised (i.e., slower reaction time or higher error rate) when having to inhibit their response in emotional “nogo” trials compared to the other age groups. The slowing effect of the affective task in our study did not show differences as a function of sex among adolescents or adults. Evidently, the specificities of “hot” EF concerning affective inhibition and affective flexibility in adolescents require further disentanglement.

The *n*-2 Switch was included in order to test the effect of a double switch inhibitory effect observed in previous studies (ABA; Koch et al., 2010). This was not confirmed as can be seen by the non-significant *n*-1 Switch × *n*-2 Switch interaction. Indeed, switching twice in a row did not seem to impact RT in our study. This effect may not have occurred as we did not include a third task in our switching paradigm. Generally, studies that have demonstrated the inhibition effect from *n*-2 repetition costs have done so using a three-task switching paradigm (Koch et al., 2010). In this case, since there were only two tasks, task sets may have been configured as switching versus non-switching cognitive sets instead of task-specific cognitive sets. This is plausible seeing as the main effect of a *n*-2 switch is nonetheless significant, suggesting that a switch two trials before can affect the RT in a given trial, when there is an absence of a *n*-1 switch.

### Associations With the Go-Nogo

As predicted, our results reveal that, when combining the adolescent and adult sample, cognitive-affective switching is associated with the indices of the Go-Nogo, i.e., percentage of commission and omission errors as well as mean RT. These results, however, are specific to the different types of switching in this task. Specifically, when combining both samples, our results imply that higher affective to cognitive switch costs seem to be related to omission errors and cognitive to affective switch costs seem to be more related to commission errors. In other words the former may me more related to problems with inattention or vigilance and the latter may be more related problems with inhibition or impulsivity. This could partly be explained by the fact that emotional content is considered a dominant task-set and captures attention more strongly and could prove harder to suppress when responding (Reeck and Egner, 2015). Some studies have shown that omission errors are more related to inattention and commission errors to impulsivity in this type of task (Bezdjian et al., 2009; Weafer et al., 2013). However, these relationships are not always clear, with one study showing that faster commission errors are more related to impulsivity and slower commission errors are more related to inattention (Halperin et al., 1988). Other studies have only found a relationship between omission errors and inattention, but not between commission errors and impulsivity (Malloy-Diniz et al., 2007; Fields et al., 2009). Another hypothesis would be that omissions errors may be more related to problems with vigilance, as the main indicator of inattention in neurodevelopmental disorders is usually that of RT variability (Geurts et al., 2008; Willcutt et al., 2008). Overall, studies are inconsistent, indicating that further investigation is needed to be certain of these inferences.

When looking at associations in each age group separately, it would seem that switch costs may be more related to inattention in adolescents and more with inhibition in adults. In adolescents, all types of switching seem to be related to a higher percentage of omissions, suggesting that inattention or a lack of vigilance, could be the principal culprit in switching costs in this group. However, a counter-intuitive result to this interpretation, is the relationship between lower RT variability as measured by RT CV and higher switching costs in adolescents. Although these associations did not survive correction, they would suggest that, although cognitive-affective switching is more related to omission errors, it is associated negatively with RT variability. This is surprising considering that RT variability is expected as an indicator of inattention (Geurts et al., 2008; Willcutt et al., 2008). However, this could also mean that, for adolescents, switching in this task may be related more with problems with vigilance, as measured by omissions, but not inattention. Evidently, the link between affective flexibility and attention requires further study. In adults, affective flexibility, especially when switching from cognitive to affective may be more related to inhibition as can be seen by the associations with commission errors and the absence of associations with omission errors. Further investigation is needed to confirm conclusions regarding the link between affective flexibility, inattention and inhibition, by, for example, studying this construct in clinical populations specifically. What seems to be of relative certainty is the relationship between affective flexibility and differences in “cool” executive processes, suggesting that these differences could be echoed in “hot” EF processes.

### Associations With the Cognitive Emotion Regulation Questionnaire

Regarding the cognitive-affective switching and the CERQ associations, the repeated finding is a relationship between the blame-other dimension and affective to cognitive switching. However, this effect did not survive correction. One could wonder whether a larger sample size would have strengthened the effect. The effects itself could potentially be explained by the switching cost that results from inhibiting the previous affective task. Although research on blaming others is sparse, some have found that it relates to reactive aggression as opposed to pro-active aggression in adolescence (Koolen et al., 2012). Reactive aggression has been shown to be due to poor judgement of social cues and impaired emotion regulation. If we look at our results in specific age-groups, affective to cognitive switching may be related to blaming others in adolescents specifically albeit marginally. It has also been shown that blaming others relates to maladaptive suppression of anger. Maladaptive suppression of anger and reactive aggression tend to be associated with impaired EF, such as difficulties with inhibitory control and impulsivity (Frick and Viding, 2009; Poland et al., 2016; Hecht and Latzman, 2018). Regardless of the strength of our effect, investigating the effects of a cognitive-affective switching task in clinical populations with affective impulsivity and emotion regulation may, nonetheless, be interesting.

In fact, de Vries and Geurts (2012) did just this when they investigated this type of switching in children with ASD. They found that the ASD group had greater difficulty in disengaging from the affective task when switching from affective to cognitive. They argue that a cognitive-affective switching task is more ecologically valid in the context of studying flexibility in ASD, considering these individuals’ frequent difficulties with emotion regulation. Others have studied the relationships between affective switching and specific affective regulatory processes. Genet and Siemer (2011) found that higher switch costs in inconsistent trials of a cognitive-affective switching paradigm using emotional words predicted lower trait resilience scores. Trait resilience is defined as a personality trait that allows one to effectively cope and adjust themselves to adversity and has been shown to be related to effective emotion regulation (ER; Ong et al., 2006; Min et al., 2013; Hu et al., 2015). The authors argue that this association may be linked to an underlying relationship between effective ER and cognitive flexibility. However, those who have revealed a link between cognitive flexibility and ER are few. Relationships between depression and impaired cognitive flexibility, and between inhibitory set-shifting (i.e., RT cost after two consecutive switches) and ruminative tendencies have previously been documented (Cannon, 1996; Whitmer and Banich, 2007). These studies give us some information regarding the link between flexibility and ER. However, future research could focus on studying the differential relationships that affective flexibility and cognitive flexibility maintain with emotion regulation. Differentiating these constructs in the study of clinical conditions that are particularly struck by emotional dysregulation, such as Borderline Personality Disorder and ADHD, could increase our understanding of the specific processes that underpin the psychosocial difficulties these people face in daily life.

## Limitations and Conclusion

Limitations in this study mostly revolve around the sample size of the adolescent group; our regressions could have profited from a larger sample. Moreover, it could have been interesting to control for certain variables such as emotion recognition and intellectual abilities, in order to strengthen our design. Further studies should aim at including controlled variables that are pertinent with regard to the variables of interest.

This study offers a first look at the age and sex differences in a cognitive-affective switching task using unpredictable switching cues. In addition, it unravels relationships between affective flexibility and other psychological constructs, such as inattention, inhibition and cognitive-emotional coping strategies. Based on our results, we conclude that there may be a specific effect of affective flexibility in women relative to men, particularly when switching from cognitive information to affective information. Regarding the effect of age, we conclude that age-related differences in cognitive-affective switching may primarily be due to differences in “cool” EF and affective inhibition between both adolescents and adults. Our findings also suggest that affective flexibility may be related to different aspects of “cool” EF, namely attention and inhibition. Relationships with cognitive-emotional coping strategies did not survive correction. Therefore, this question remains open. Future directions could aim at examining the relationship between “hot” EF and specific ER processes. Understanding these constructs more precisely could allow for an improved integration of cognitive and affective processes in research. Considering all findings as a whole, affective flexibility is undeniably tied to “cool” EF processes. However, affective flexibility can also be subject to differences related to the processing of affective processes specifically, as is evident from the sex differences we found in this study. This effect is particularly important since we know that females tend to process affective stimuli differently than males. Thus, one wonders how others population-based cohorts, whether demographic or clinical, that show specific differences in emotional processing as well would perform when being tested by this task.

## Data Availability Statement

The raw data supporting the conclusions of this article will be made available by the authors, without undue reservation.

## Ethics Statement

The studies involving human participants were reviewed and approved by Ethics Committee of the Department of Psychology and Educational Sciences of the University of Geneva, Switzerland. Written informed consent to participate in this study was provided by the participants’ legal guardian/next of kin.

## Author Contributions

JS contributed to the methodology, formal analysis, data curation, writing – original draft, writing – reviewing and editing, and visualisation. LR contributed to the conceptualisation, software, methodology, and writing – reviewing and editing. JC contributed to the methodology, validation, writing – reviewing and editing, and formal analysis. DB contributed to the conceptualisation, software, project administration, methodology, and data curation. NP contributed to the supervision, and writing – reviewing and editing. MD contributed to the conceptualisation, supervision, funding acquisition, writing – reviewing and editing, and methodology. All authors contributed to the article and approved the submitted version.

## Conflict of Interest

The authors declare that the research was conducted in the absence of any commercial or financial relationships that could be construed as a potential conflict of interest.

## Publisher’s Note

All claims expressed in this article are solely those of the authors and do not necessarily represent those of their affiliated organizations, or those of the publisher, the editors and the reviewers. Any product that may be evaluated in this article, or claim that may be made by its manufacturer, is not guaranteed or endorsed by the publisher.
